# Implementation and Outcomes of the New York State YMCA Diabetes Prevention Program: A Multisite Community-Based Translation, 2010–2012

**DOI:** 10.5888/pcd11.140006

**Published:** 2014-07-10

**Authors:** Anne Bozack, Susan Millstein, Jacqueline Martinez Garcel, Kim Kelly, Rachael Ruberto, Linda Weiss

**Affiliations:** Author Affiliations: Susan Millstein, Kim Kelly, Rachael Ruberto, New York State Department of Health, Albany, New York; Jacqueline Martinez Garcel, New York State Health Foundation, New York, New York; Linda Weiss, The New York Academy of Medicine, New York, New York.

## Abstract

**Introduction:**

Weight loss and physical activity achieved through the Diabetes Prevention Program (DPP) have been shown to reduce type 2 diabetes risk among individuals with prediabetes. The New York State Young Men’s Christian Association (YMCA) delivered the 16-week evidence-based model at 14 YMCAs. A mixed methods process and outcomes evaluation was conducted.

**Methods:**

Most participants were referred by clinicians and were encouraged to achieve 5% to 7% weight loss. Participants were weighed weekly; additional data were gathered from participant surveys and focus groups and staff surveys and interviews.

**Results:**

Participants (N = 254) lost a mean of 9 pounds (*P* < .001), or 4.2% of body weight, by program completion; 40% achieved 5% or more weight loss and 25% achieved 7% or more weight loss. Ten months after baseline, 61% of participants reported 5% or more weight loss and 48% reported 7% or more weight loss. In multivariate models, weight loss was negatively associated with black race (16 weeks: adjusted odds ratio [AOR], 0.190, *P* = .002; 10 months: AOR, 0.244, *P* = .005) and positively associated with attendance (16 weeks: AOR, 18.699, *P* < .001; 10 months: AOR, 2.808, *P* = .024). Participants reported improvements in health and lifestyle changes after program completion. Factors contributing to program success included coaches who motivated participants, the group setting, curriculum, and program duration. However, sociodemographic diversity was limited.

**Conclusion:**

Outcomes demonstrate the potential for effectively implementing the DPP in community-based settings. Findings also suggest the need for replications among a broader population.

## Introduction

Diabetes is a chronic disease of growing concern in the United States. It is the leading cause of kidney failure, nontraumatic lower limb amputations, and adult-onset blindness (1), and type 2 diabetes reduces life expectancy by 6 to 10 years (2,3). Type 2 diabetes can develop from prediabetes (4), a condition characterized by glucose levels above normal but not meeting the criteria for diabetes. Approximately one-third (1) of American adults have prediabetes, 5% to 10% of whom will progress to type 2 diabetes annually (5,6).

The Diabetes Prevention Program (DPP), a randomized controlled trial of a lifestyle change program implemented from 1996 through 2002 with up to 4.6 years of follow-up, demonstrated that individuals with prediabetes who achieved a 5% to 7% weight reduction and participated in regular physical activity reduced their diabetes risk between 58% and 71%, twice that achieved through medication management (7). To enhance the feasibility of widespread adoption, the Indiana University School of Medicine partnered with a Young Men’s Christian Association (YMCA) site from 2004 through 2006 to implement and evaluate the DPP as a group and community-based program known as Diabetes Education and Prevention with a Lifestyle Intervention Offered at the YMCA (DEPLOY). Participants lost an average of 6% of their body weight, consistent with the original trial (8).

DEPLOY has been translated through other YMCAs and faith- and community-based settings (9–11) with generally positive outcomes. The literature on these replications focuses on weight loss and clinical markers with limited data on general health or changes in knowledge and behavior, which are relevant to sustained weight loss (12,13). Similarly, there is a dearth of information on factors affecting implementation, despite the utility for translation in additional settings. In an effort to address these gaps in the literature, we describe results from the implementation and evaluation of the DEPLOY model in YMCAs in New York State (NYS) from 2010 through 2012, known as the NYS YMCA Diabetes Prevention Program (Y-DPP). Findings are drawn from a qualitative and quantitative process and outcome evaluation, and they include information on participant health, knowledge and behavior, staffing needs, and program perceptions.

## Methods

### The New York State YMCA Diabetes Prevention Program

The NYS Department of Health’s Diabetes Prevention and Control Program (DPCP) convened a statewide advisory group to develop the Y-DPP, including recruitment materials and a physician referral algorithm. The DPCP also facilitated training and ongoing technical assistance to Y-DPP coordinators and lifestyle coaches. The Y-DPP used the group-based DEPLOY curriculum, which focuses on improved nutrition and increased physical activity, and consists of 16 weekly core sessions and 6 monthly maintenance sessions led by trained coaches (8). Participants are encouraged to achieve 5% to 7% weight loss by the end of the core sessions. Although implemented before the National DPP of the Centers for Disease Control and Prevention (CDC) was established, the Y-DPP was largely consistent with the National DPP curriculum (14) and recognition standards (15). The Y-DPP was implemented through the Alliance of New York State YMCAs at 14 YMCA sites serving urban, suburban, and rural communities. It was provided without charge to participants, who also received free, limited-time YMCA membership. Implementation was funded by the New York State Health Foundation.

### Recruitment and eligibility

The Y-DPP was unique in recruiting participants through clinical–community linkages. DPCP staff conducted in-person outreach to health care providers, some of whom had existing partnerships with the YMCAs, and developed relationships with more than 30 providers who referred patients to the program. Participants also learned about the program though YMCA outreach, media, and word of mouth. Individuals were eligible if they were aged 18 years or older and provided a physician referral form indicating diabetes risk. Determination of diabetes risk was based on physician discretion, but physicians were encouraged to use the Y-DPP referral algorithm (ie, body mass index [BMI] ≥25 kg/m^2^ and at least 1 of the following: fasting plasma glucose 100–125 mg/dL, 2-hour plasma glucose 140–199 mg/dL, random capillary blood glucose 110–199 mg/dL, or hemoglobin A1c 5.7%–6.4%), similar to the CDC’s National DPP recognition standards for blood-based diagnostic tests indicating prediabetes (15). Individuals were ineligible if they had been diagnosed with diabetes.

Participation in the evaluation was voluntary and did not affect eligibility for the Y-DPP. The evaluation protocol and instruments were approved by The New York Academy of Medicine Institutional Review Board. Individuals who agreed to participate in the evaluation provided signed informed consent.

### Evaluation design

#### Participant-level data

Participant height was obtained from physician referral forms. Coaches weighed participants and recorded weight, attendance, and self-reported physical activity at each core session. During the first class, participants completed baseline surveys with questions on demographics, health status (ie, “In general, would you say your health is excellent, very good, good, fair, or poor?”) (adapted from the Behavioral Risk Factor Surveillance System questionnaire [16]), health-related quality of life (EuroQol EQ-5D-3L [17]), challenges to healthy behaviors, and referral source. Follow-up surveys covered similar topics as well as changes in health knowledge and behavior and satisfaction with the Y-DPP. Sixteen-week surveys were completed during the last Y-DPP class. If a participant did not attend the last class, evaluation staff administered the survey by telephone. A second follow-up was completed by telephone approximately 6 months after program completion (ie, 10 months from baseline), and participants were asked to self-report their weight to the nearest pound. The survey was mailed to participants who could not be contacted by telephone. Participants received a $10 gift card upon completing the 10-month survey.

Focus groups were conducted at 3 sites with participants within 1 week of their completing the core sessions or participants who were currently engaged in the monthly maintenance sessions. Coaches invited participants to attend the appropriate focus groups. Questions addressed the referral process, program components, health, health behaviors, perceptions, and recommendations. Evaluation staff experienced with qualitative research methods facilitated the focus groups.

#### Program-level data

At the end of each core session, coaches completed surveys on program fidelity, participant engagement, time commitment, perceptions, and recommendations. Coordinators at each branch completed surveys on YMCA characteristics, time commitments, participant incentives, and recruitment strategies. Semistructured individual or group interviews were conducted with coaches, coordinators, and other participating staff members at 3 YMCAs. Questions were similar to those in coach and coordinator surveys.

#### Analytical methods

Survey data were maintained by using Microsoft Office Access version 2007 (Microsoft Corp, Redmond, Washington) and analyzed using IBM SPSS version 19 (IBM Corp, Armonk, New York). Analyses of staff surveys were limited to descriptive statistics because of small sample sizes. Means and proportions were generated for baseline participant characteristics. Preanalyses and postanalyses were conducted for changes in weight and health status. To minimize bias, follow-up values for participants with missing 16th-week in-class weight were determined by carrying forward the last value recorded. Ten-month weight relied on self-report. Weight change from baseline was analyzed by participant demographic characteristics and attendance. Attendance was dichotomized into 1 to 8 sessions and 9 or more sessions, consistent with the CDC’s National DPP recognition standards (15). Mean changes in weight were analyzed using paired *t* tests. Demographic variables and variables attaining significance at the *P* < .1 level in bivariate analyses were entered into multivariate models. Change in health status from baseline was analyzed using Stewart-Maxwell and McNemar’s tests. Means and proportions were calculated for knowledge and behavioral changes.

Participant focus groups and staff interviews were audiorecorded; focus groups were transcribed and detailed notes were taken during the interviews. Transcriptions and notes were maintained, coded, and analyzed in NVivo version 8 (QSR International, Doncaster, Victoria, Australia). They were coded using preidentified themes consistent with evaluation questions and using themes emerging from the data.

## Results

### Participant characteristics

The evaluation had 254 participants representing 26 courses implemented during October 2010 through May 2011. Most participants had a BMI of 25 or higher and a clinical diagnosis of prediabetes (78.7%); 57.5% had learned about the Y-DPP from a clinical provider (Table 1). Follow-up rates for the 16-week and 10-month surveys were 85.4% and 76.8%, respectively.

**Table 1:** Characteristics of Participants in the New York State YMCA Diabetes Prevention Program, 2010–2012

Characteristic (N = 254)	Value^a^
**Female**	178 (70.1)
**Age, y, mean (SD)**	57.4 (11.3)
**Race/ethnicity^b^**	
White	197 (77.9)
Black	37 (14.6)
Hispanic	9 (3.6)
Other	10 (4.0)
**Highest level of education**	
High school graduate or below	54 (21.3)
Some college or vocational school	85 (33.5)
College graduate	115 (45.3)
**Employment status^c^**	
Employed full-time or part-time	139 (55.8)
Not working or unable to work	32 (12.9)
Retired	86 (34.5)
**Annual household income, $^b^**	
<40,000	65 (28.4)
40,000–79,999	84 (36.7)
≥80,000	80 (34.9)
**Residence^b^**	
Suburban	100 (42.2)
Urban	71 (30.0)
Rural	66 (27.8)
**Health insurance^c^**	
Private	195 (77.7)
Medicare	71 (28.3)
Medicaid	16 (6.4)
Veterans Affairs	4 (1.6)
None	9 (3.6)
**Baseline weight, lb, mean (SD)**	214.2 (50.9)
**Baseline body mass index, kg/m^2^, mean (SD**)	35.0 (7.9)
**Y-DPP referral source**	
Doctor or other heath provider	142 (57.5)
YMCA brochure or website	54 (21.9)
Family or friend	30 (12.1)
News report	17 (6.9)
Other	4 (1.6)

Abbreviation: SD, standard deviation; YMCA, Young Men’s Christian Association; Y-DPP, YMCA Diabetes Prevention Program.

a Data are no. (%) unless otherwise specified.

b N < 254 because of missing responses. Missing values were excluded from calculations.

c Multiple responses were possible.

Participants were predominantly women (70.1%) and white (77.9%), had an annual household income of $40,000 or more (71.6%), had private health insurance (77.7%) (Table 1), were aged 45 years or older (87.4%, range, 24–84; mean, 57), and had a primary care provider (98.4%).

Participants who completed the follow-up surveys were compared with those who did not by age, sex, race, education, income, and insurance status. Compared with participants who completed the 16-week survey, participants who did not complete the survey were younger, more likely to be black, less likely to be white, and had less education and lower income (*P* < .05). Participants who did not complete the 10-month survey were younger and had lower income than those who did complete the survey (*P* < .05).

Three focus groups were conducted with individuals within 1 week of completing the core sessions (N = 18; group size ranged from 5 to 7) and 3 groups were conducted with individuals engaged in the maintenance sessions (N = 19; group size ranged from 4 to 9). Focus group participants were demographically similar to all program participants — most were women (64.9%), white (86.5%), and aged 45 or older (88.9%; range, 30–82; mean, 61).

### Participant outcomes

Participants attended an average of 10.6 of the 16 sessions; 72.3% attended more than half of the sessions (ie, 9 or more sessions). In surveys, the most commonly noted motivations for participation were worry about diabetes or prediabetes (70.2%) and weight loss (63.2%). Focus group findings were consistent; participants were aware of the health implications of diabetes, often because of family history, and wanted to avoid medication dependence.

My mom died a year ago of complications of diabetes. . . . [I] saw firsthand what this disease does to you — toes amputated and heart problems. [Female, Site A, Core sessions completion group]

Sixteenth-week weight was imputed for 24.4% of participants, and 10-month weight was obtained through self-report from 74.4% of participants. At program completion, participants lost a mean of 9.0 pounds (*P* < .001), or 4.2% of body weight (Table 2); 40.2% achieved 5% or greater weight loss, and 24.8% achieved 7% or greater weight loss. At 10 months, 60.8% reported 5% or greater weight loss, and 47.6% reported 7% or greater weight loss.

**Table 2:** 16-week and 10-Month Weight Loss by Sex, Age, and Attendance, New York State YMCA Diabetes Prevention Program, 2010–2012

Characteristic	N	Pounds Lost, Mean (SD)	*P* Value^a^	Percentage of Body Weight Lost, Mean (SD)	Loss of ≥5% Body Weight, n (%)	Loss of ≥7% Body Weight, n (%)
**16-week weight loss**						
**All participants**	254	9.0 (9.9)	<.001	4.2 (4.6)	102 (40.2)	63 (24.8)
**Attendance**						
1–8 classes	68	2.3 (5.0)	<.001	1.0 (2.1)	5 (7.4)	1 (1.5)
9–16 classes	186	11.4 (10.1)	<.001	5.4 (4.7)	97 (52.2)	62 (33.3)
**Sex**						
Female	178	7.4 (8.4)	<.001	3.6 (3.9)	65 (36.5)	38 (21.3)
Male	76	12.8 (11.9)	<.001	5.7 (5.6)	37 (48.7)	25 (32.9)
**Age, y**						
<45	32	6.9 (10.0)	<.001	3.1 (4.7)	11 (34.4)	6 (18.8)
45–54	56	7.4 (7.6)	<.001	3.4 (3.5)	18 (32.1)	9 (16.1)
55–64	93	10.2 (10.6)	<.001	4.9 (4.9)	41 (44.1)	29 (31.2)
≥65	73	9.4 (10.3)	<.001	4.6 (4.6)	32 (43.8)	19 (26.0)
**Race/ethnicity**						
White	197	9.8 (10.3)	<.001	4.7 (4.7)	89 (45.2)	56 (28.4)
Black	37	6.0 (8.0)	<.001	2.5 (3.5)	8 (21.6)	3 (8.1)
Hispanic	9	5.8 (6.0)	.02	3.4 (3.6)	3 (33.3)	2 (22.2)
Other	10	4.8 (7.0)	.06	2.4 (3.2)	1 (10.0)	1 (10.0)
**10-month weight loss^b^**						
**All participants**	189	13.3 (15.4)	<.001	6.3 (6.7)	115 (60.8)	90 (47.6)
**Attendance**						
1–8 classes	34	5.6 (13.8)	.03	2.7 (6.4)	14 (41.2)	12 (35.3)
9–16 classes	155	15.0 (15.2)	<.001	7.0 (6.5)	101 (65.2)	78 (50.3)
**Sex**						
Female	127	11.6 (14.8)	<.001	5.6 (6.5)	73 (57.5)	55 (43.3)
Male	62	16.8 (16.1)	<.001	7.6 (6.9)	42 (67.7)	35 (56.5)
**Age, y**						
<45	20	12.4 (16.3)	.003	5.6 (7.3)	11 (55.0)	9 (45.0)
45–54	37	10.1 (11.2)	<.001	4.7 (5.1)	19 (51.4)	16 (43.2)
55–64	69	13.2 (17.9)	<.001	6.3 (7.7)	39 (56.5)	31 (44.9)
≥65	63	15.6 (14.0)	<.001	7.3 (6.2)	46 (73.0)	34 (54.0)
**Race/ethnicity**						
White	148	14.9 (15.1)	<.001	7.0 (6.5)	98 (66.2)	78 (52.7)
Black	26	5.0 (17.1)	.15	1.9 (7.2)	8 (30.8)	6 (23.1)
Hispanic	7	9.8 (7.1)	.01	5.4 (4.3)	4 (57.1)	3 (42.9)
Other	8	13.9 (13.1)	.02	6.8 (5.8)	5 (62.5)	3 (37.5)

Abbreviation: SD, standard deviation.

a Paired *t* test for baseline and follow-up weight (lbs).

b Based on self-report.

Sex, age (continuous), race, education, income, and attendance were included in multivariate analyses for outcomes at 16 weeks; sex, age, race, insurance, and attendance were included for outcomes at 10 months. Age had a slight negative association with achieving 5% or more weight loss at 16 weeks (adjusted odds ratio [AOR], 0.96; 95% CI, 0.94–0.996; *P* = .03). At both 16 weeks and 10 months, black race was negatively associated (AOR, 0.19, 95% CI, 0.07–0.54, *P* = .002, and AOR, 0.24, 95% CI, 0.09–0.65, *P* = .005, respectively) and attendance at more than half of the core sessions was positively associated (AOR, 18.70, 95% CI, 6.55–53.41, *P* < .001, and AOR, 2.81, 95% CI, 1.15–6.86, *P* = .02, respectively) with 5% or more weight loss.

Most participants reported improvements in overall health. The proportion of participants reporting very good or excellent health increased significantly from 31% at baseline to 44% at 16 weeks and 56% at 10 months (*P* < .001) (Table 3). At 10 months, participants reported fewer problems with mobility (*P* = .011), pain (*P* = .001), and performing usual activities (*P* = .011), compared with baseline.

**Table 3:** Self-Reported Health Status at Baseline, 16 Weeks, and 10 Months, New York State YMCA Diabetes Prevention Program, 2010–2012

Characteristic	Baseline (N = 254)	16 weeks (n = 217)		10 months (n = 195)	
	n (%)	n (%)	*P* value^a^	n (%)	*P* value^a^
**General health^b^^,^ c**					
Excellent or very good	79 (31.2)	96 (44.2)	<.001	109 (56.2)	<.001
**Problems^b^**					
Mobility	58 (22.9)	53 (24.4)	.49	32 (16.4)	.01
Self-care	5 (2.0)	9 (4.2)	.03	8 (4.1)	.22
Usual activities	52 (20.6)	37 (17.1)	.37	25 (12.8)	.01
Pain	152 (61.3)	111 (51.4)	.01	90 (46.2)	.001
Anxiety or depression or both	66 (26.8)	61 (28.2)	.88	43 (22.1)	.63

a McNemar’s test.

b Missing values were excluded from calculations.

c General health categories were excellent, very good, good, fair, and poor.

Focus group respondents reported similar improvements in health status, attributing improvements to weight loss and physical activity.

The difference was like night and day, as far as my energy level and my mood and everything else. . . . I started to feel healthy and act healthy. [Female, Site B, Maintenance sessions group]

I had some back problems. . . . I did the program and I lost some weight. That really helped. [Male, Site A, Maintenance sessions group]

Participants were asked to compare their baseline and current knowledge and behaviors in measuring portion sizes; eating grains, fruits, vegetables, and fat; making healthy choices when eating out; and avoiding social cues that cause unhealthy eating. At program completion and at 10 months, almost all participants reported improvements in these areas and the intention to continue healthy choices, although there was a downward trend from program completion to 10 months. Qualitative findings provide more detail:

I've become more aware of food portions in weight and liquid form and calories. . . . My wife started calling me a born-again nutritionist. [Male, Site A, Core sessions completion group]

I used to leave things at the bottom of the stairs and whoever went up next, took them. Now I just make a trip up. . . . It was encouraged in the class to do that extra. [Female, Site B, Core sessions completion group]

### Program implementation and perceptions

Each YMCA had a program coordinator and 1 or more coaches who were generally current staff. Program coordination, including outreach and recruitment, required from 2 to 20 hours of staff time per week (median, 7.0 hours). Coach time per class varied between coaches from 2 to 10 hours per week (median, 4.0 hours), including class preparation, instruction, and support for participants outside of class. At most branches, other staff members or additional coach time was necessary to support publicity, recruit participants, engage clinical partners, and perform administrative activities.

Overall, perceptions were positive, even among participants who did not complete the program. A minority of participants and coaches expressed concern about the curriculum’s emphasis on fat reduction, with relatively little information on carbohydrates, sugar, and sodium. An earlier introduction to physical activity was also suggested, to take advantage of early motivation of participants and access to YMCA facilities. On the positive side, the YMCA was considered to be an appropriate setting, described as a “healthy” and motivational environment.

All coaches were available either by telephone or at the YMCA to assist participants outside of scheduled sessions. Participants emphasized the coach’s integral role in providing motivation and guidance for making lifestyle changes. Participants and YMCA staff felt that coaches had knowledge and ability to address issues related to diet, exercise, social and psychological factors that may affect the ability to make lifestyle changes, and the skills and experience necessary to encourage group interaction.

The success of the program . . . in a large part is due to [the coach’s] knowledge, his competence, . . . [and his] background in physical training, exercise, and nutrition. . . . He’s taken quite numerous participants over the hump with things that stop them from proceeding. [Male, Site A, Core sessions completion group]

[YMCA staff members] have gone through so much [training] as far as the active listening, the relationship-building. . . . [Getting] the whole group to participate is critical. [Site B, Staff interview]

Numerous participants reported that the group setting provided social support and a forum to exchange ideas. Participants also felt accountable to the larger group and were therefore more likely to attend sessions and maintain lifestyle changes.

[The group] was very supportive. . . . [Members] were struggling or hitting obstacles that all of us experience. . . . [The group provided] information about how other people are hitting some of these roadblocks. [Male, Site A, Core sessions completion group]

However, negative perceptions of the groups were also reported, focusing on limited sociodemographic diversity or feelings of noninclusion. For example,

I wish it had been geared toward people of a lower economic strata. It was like everyone in the class was in a different world than I was. [Female, Site C, Survey response]

Participants generally found the length of the Y-DPP to be appropriate, enabling them to change habits and form healthy routines. Participants were more likely to suggest a longer rather than a shorter program or more sessions during the maintenance period.

By the end of 4 months it’s now a norm for me and instead of being unusual, it’s hard for me to think about eating a different way. [Male, Site A, Core sessions completion group]

## Discussion

The Y-DPP demonstrated the feasibility and advantages of translating the group-based DPP model to a community-based setting by using existing resources: clinicians with existing linkages with YMCAs; YMCA fitness facilities; and YMCA staff members, who had prior experience implementing wellness programs. Furthermore, this study expands on previous research by demonstrating effects on general health, quality of life, and knowledge and behavior, and providing findings important for program replication, including staff time commitments and participant and coach perspectives.

Consistent with other research (8–10), our study provides evidence for the effectiveness of the DPP model to promote weight loss. By the end of the program, 40% of participants lost 5% or more body weight and 25% lost 7% or more — levels associated with a reduction in type 2 diabetes risk (7). Mean weight loss was 4.2%, comparable to the 3.99% reduction reported in a meta-analysis of DPP translation studies (9). Most participants maintained weight loss or continued to lose weight after the core sessions; 60% reported 5% weight loss 6 months after program completion.

Overall, the program was well-received among participants and coaches. Contrary to common concerns, participants felt that a relatively lengthy program was necessary for changing habits. Similarly, the positive association between attendance and weight loss and slight decrease in lifestyle changes reported 6 months after program completion suggests the importance of program length and ongoing support. Minor concerns regarding the curriculum focused on the emphasis on fat and the late introduction of physical activity; the rationale behind these practices should be explained to participants and coaches.

Other concerns focused on the program’s suitability for low-income and minority participants. Findings indicate lower levels of engagement, and although numbers were too small for definitive comparisons, data suggest the possibility of poorer outcomes. Qualitative findings indicate that low-income and minority participants may not have felt a connection or sense of inclusion within the groups. Given the prevalence of diabetes among these populations, such findings suggest the need for targeted recruitment and possible program adaptations.

This study had several limitations. Participation in the program and evaluation were voluntary; therefore, results may have differed for less motivated populations. Insufficient data were available regarding the suitability and effectiveness of the Y-DPP among some sociodemographic groups with low enrollment rates, including minorities, low-income individuals, and individuals who are underinsured or uninsured. Although demographic characteristics of people who were referred but did not enroll are unknown, recruitment through clinical provider referrals likely limited enrollment among low-income and minority individuals, who are less likely to have a regular source of care (18,19). In addition, compared with participants who completed follow-up surveys, participants who did not complete follow-up surveys were younger, more likely to be black, less likely to be white, and had less education and lower income, possibly affecting the generalizability of findings.

Inclusion of a control group was not feasible because of resource constraints and determined to be inappropriate given the existing evidence of the program’s efficacy. Quantitative data were limited to preanalysis and postanalysis information collected by the YMCA and through surveys. Although most participants had a clinical diagnosis of prediabetes, follow-up clinical indicators of diabetes risk were not available. In addition, 16th-week weights were imputed for participants with missing in-class weight data (24%), and all 10-month weights relied on self-report. Follow-up data were also limited to 6 months after program completion; an extended follow-up period is necessary to assess the long-term sustainability of lifestyle changes.

Despite these limitations, this article makes an important contribution to the literature, because of the scope of the Y-DPP and its evaluation. As insurers and policy makers consider reimbursement for disease-prevention programs as a cost-effective alternative to clinical treatment, comprehensive evaluations of programs implemented under a range of conditions are crucial. Although this study was implemented before CDC’s National DPP recognition standards were established, implementation was largely consistent with current standards and, therefore, findings contribute to the literature supporting the expansion of evidence-based National DPP programs in community-based settings. The YMCA has the unique advantage of on-site fitness facilities, experienced staff, and connections to clinical providers; other organizations may need to consider their capacity in these areas before replication. Findings also suggest the critical need for targeted approaches to reach and retain a broader population, including men, minorities, and individuals who are low-income and uninsured or publicly insured.
